# Pretreatment emotional distress and peripheral biomarkers predict immune checkpoint inhibitor response in people with advanced inoperable gastroesophageal cancer

**DOI:** 10.1038/s43856-025-01358-9

**Published:** 2026-01-26

**Authors:** Runze Huang, Guodong Nie, Anlong Li, Xueting Ding, Mengqian Liu, Ling Cheng, Senbang Yao, Han Ge, Jiaying Chai, Yingxue Jia, Lijun Liu, Zhonglian Huang, Huaidong Cheng, Mingjun Zhang

**Affiliations:** 1 School of Medicine, Tongji University, Shanghai, China; 2 Department of Medical Oncology, Shanghai Pulmonary Hospital, Shanghai, China; 3 Department of Oncology, the Second Affiliated Hospital of Anhui Medical University, Hefei, Anhui China; 4 Gamma Knife Unit, Tumor Center, Huainan Chaoyang Hospital, Huainan, Anhui China; 5 Anhui Medical University, Hefei, Anhui China; 6 Department of Health, Society, & Behavior, Joe C. Wen School of Population & Public Health, Susan and Henry Samueli College of Health Sciences, University of California, Irvine, Irvine, CA USA; 7 Department of Nephrology, the Second Affiliated Hospital of Anhui Medical University, Hefei, Anhui China; 8 Department of Oncology, Shenzhen Hospital of Guangzhou University of Chinese Medicine (Futian), Shenzhen, China; 9 School of Nursing, Anhui Medical University, Hefei, Anhui China; 10 The Third School of Clinical Medicine, Southern Medical University, Guangzhou, China; 11 Department of Oncology, Shenzhen Hospital of Southern Medical University, Shenzhen, Guangdong China

**Keywords:** Tumour biomarkers, Gastrointestinal cancer, Cancer immunotherapy

## Abstract

**Background:**

Emotional distress (ED) has been demonstrated to compromise immune responses against tumors; however, few clinical studies have explored its influence on the efficacy of immune checkpoint inhibitors (ICIs) in cancer patients, especially those with gastroesophageal cancer (GEC). Additionally, reliable biomarkers for predicting the response to immunotherapy remain elusive. This study was aimed at investigating whether ED affects the outcomes of immunotherapy in advanced GEC patients and identifying potential biomarkers predictive of immunotherapy efficacy.

**Methods:**

This prospective observational cohort study enrolled 84 patients with advanced, treatment-naïve, and inoperable GEC. ED was evaluated at baseline using the Patient Health Questionnaire-9 and the Generalized Anxiety Disorder 7-item Scale. The primary endpoint was Progression-Free Survival (PFS), while the secondary endpoint was Disease Control Rate (DCR).

**Results:**

Patients with baseline ED exhibit significantly shorter median PFS (7.8 months vs. 14.0 months, HR = 2.59, 95% CI: 1.35-4.97, *P* = 0.004) and a lower DCR (39.5% vs. 68.3%, OR = 3.21, 95% CI: 1.29–7.98, *P* = 0.012) compared to those without ED. Exploratory analyses further demonstrate that both pre- and post-treatment peripheral inflammatory markers (PIMs) are independently and jointly associated with survival outcomes in combination with ED.

**Conclusions:**

This prospective study demonstrates that ED and elevated PIMs significantly impair ICI efficacy in advanced GEC. The synergistic interaction between ED and PIMs suggests underlying psycho-inflammatory mechanisms affecting treatment outcomes. These findings establish the clinical importance of integrating routine psychological assessment and PIMs monitoring in cancer patients receiving immunotherapy.

## Plain language summary

This study explored how stress and inflammation affect treatment outcomes in patients with advanced stomach or esophageal cancer receiving a type of treatment called immunotherapy. We aimed to understand why some patients respond better to these drugs and whether emotional health plays a role. We followed 84 patients, using questionnaires and blood tests to measure stress and inflammation before treatment. Patients with high stress before treatment saw faster cancer progression (7.8 vs. 14 months) and poorer responses to immunotherapy. High inflammation levels further worsened outcomes, especially when combined with stress. These results highlight that reducing stress and monitoring inflammation before treatment could help doctors tailor immunotherapy plans, potentially improving survival and quality of life for cancer patients.

## Abstract (web summary)

Huang et al. examine the impact of emotional distress on immune checkpoint inhibitor efficacy in advanced gastroesophageal cancer patients. Baseline emotional distress and elevated peripheral inflammatory markers significantly impair treatment outcomes through synergistic psycho-inflammatory mechanisms.

## Introduction

Gastroesophageal cancer (GEC), which includes gastric, esophageal, and gastroesophageal junction (GEJ) cancers, remains one of the leading causes of cancer-related mortality worldwide^1^. Most patients are diagnosed at advanced stages when the disease is inoperable, leaving systemic therapies as the mainstay of treatment^2^. Immune checkpoint inhibitors (ICIs), targeting programmed death-1 (PD-1)/programmed death-ligand 1 (PD-L1), have emerged as a cornerstone therapy in advanced gastroesophageal cancer. Clinical trials, such as CheckMate 649, KEYNOTE-061, and KEYNOTE-590, have demonstrated that ICIs, either as monotherapy or combined with chemotherapy, significantly improve patient outcomes compared to traditional treatments^3–5^. However, factors influencing the heterogeneous response to ICIs in gastroesophageal cancer remain incompletely understood. Although some patients exhibit no response or develop resistance, only a minority achieve long-term benefits from ICI treatment^6^. It is imperative to identify the mechanisms of heterogeneous responses and “precisely” predict treatment efficacy. Current biomarkers, such as PD-L1 expression and tumor mutation burden (TMB), have limited accuracy in predicting patient responses to ICI treatment^7^. Additionally, they also face practical challenges, including difficulties in acquisition and high associated costs^8^. Identifying reliable and accessible clinical or biological markers that accurately predict immunotherapy efficacy is crucial for developing personalized treatment strategies.

In recent years, the influence of psychosocial factors on cancer clinical outcomes has garnered increasing attention. Emotional distress (ED), which encompasses symptoms of depression and anxiety, is highly prevalent in cancer patients, with a prevalence rate approximately four times higher than in the general population^9^. Notably, ED is particularly common in GEC patients. Studies have shown that 50% of postoperative esophageal cancer patients experience symptoms such as worry, anxiety, and depression^10^, while 33.6% of patients across all stages of gastric cancer report significant levels of distress^11^. This prevalence is even higher among patients with advanced-stage GEC^12^.

Importantly, these emotional distresses are frequently associated with adverse oncological outcomes. Biologically, ED may serve as a chronic stressor promotes dysregulation of the hypothalamic-pituitary-adrenal (HPA) axis and chronic activation of the sympathetic nervous system (SNS), generating numerous stress-associated immunomodulatory molecules (SAIMS), resulting in a suppressive immune microenvironment that favors tumor growth and metastasis^13^. Preclinical studies have demonstrated that chronic stress can activate the HPA axis to increase cortisol production, leading to alterations in cytokine profiles and T cell exhaustion^14–16^. Stress also enhances anti-apoptotic mechanisms, proliferative activity, treatment resistance, and stem cell-like characteristics of tumor cells through SNS activation and increased catecholamine levels^17,18^. Multiple clinical observational studies have demonstrated that cancer patients with distress symptoms (including depression and anxiety symptoms) exhibit poorer survival outcomes and elevated mortality rates^19,20^. The aforementioned evidence suggests that ED may influence the efficacy of ICIs, though direct clinical evidence in cancer populations is lacking. However, current clinical practice lacks recognition of the importance of ED and its routine monitoring and assessment^21^. As immunotherapy is increasingly becoming the cornerstone of GEC treatment, it is imperative to investigate the association between ED and the efficacy of ICIs in GEC patients.

In addition to psychosocial factors influencing treatment outcomes, emerging evidence highlights systemic inflammation’s role in modulating ICI efficacy. Peripheral inflammatory markers (PIMs), including the neutrophil-to-lymphocyte ratio (NLR), platelet-to-lymphocyte ratio (PLR), systemic immune-inflammation index (SII), pan-immune-inflammation value (PIV), and prognostic nutritional index (PNI), are increasingly recognized as simple and cost-effective indicators of systemic inflammation and immune dysfunction in cancer patients^22,23^. These markers are easily derived from routine blood tests and have been associated with immune exhaustion, tumor progression, and treatment resistance in various solid tumors^24,25^. Emerging evidence supports the role of PIMs in stratifying patients who may benefit from ICIs, but prospective studies exploring their prognostic significance in GEC remain sparse. Furthermore, existing research has demonstrated a bidirectional relationship between ED and systemic inflammation^26^. ED could exacerbate systemic inflammation and elevate levels of PIMs^27^. Conversely, systemic inflammation plays a crucial role in the pathophysiology of depression^28^. However, it remains unclear whether ED and systemic inflammation, as indicated by PIMs, can independently or synergistically influence the efficacy of ICI treatment. Therefore, further exploration is warranted to elucidate both the individual and interactive effects of ED and PIM levels on the outcomes of cancer ICI therapy. In addition, the levels of peripheral nutritional or metabolic markers, such as serum albumin, can modulate host immune function^29^, potentially impacting the efficacy and prognosis of cancer ICI treatment. The severity of ED may also be correlated with these marker levels^30^. Thus, we monitored these peripheral metabolic markers alongside PIMs as peripheral biomarkers.

This study aims to examine these two important questions: (1) whether pretreatment ED impacts survival outcomes in patients treated with ICIs for advanced, treatment-naïve GEC; (2) whether peripheral biomarkers measured at baseline or after initial treatment can independently or in combination with ED predict the efficacy of immunotherapy. The former constitutes the primary analysis, while the latter serves as an exploratory analysis. We demonstrate that baseline ED correlates with significantly shorter PFS and reduced DCR, while elevated post-treatment PLR and other inflammatory markers exacerbate poor outcomes in patients with ED. Crucially, we identify a synergistic interaction between ED and systemic inflammation, revealing psycho-immunological mechanisms as drivers of therapeutic resistance. These findings underscore the clinical urgency of integrating psychological assessments and inflammatory markers monitoring to optimize immunotherapy in GEC. To the best of our knowledge, this is the first prospective cohort study to comprehensively evaluate the independent and combined effects of psychological and metabolic or inflammatory markers on the prognosis of patients with GEC receiving ICIs, providing actionable insights for integrating psychosocial assessments and biomarker monitoring into personalized immunotherapy strategies.

## Methods

### Study design

This prospective observational cohort study aimed to investigate the association between pre-treatment ED and the prognosis of patients with advanced, inoperable, treatment-naïve gastroesophageal cancer receiving first-line ICI treatment. Additionally, we aimed to explore peripheral blood metabolic or inflammatory markers that could serve as predictive indicators for immunotherapy response. The former constitutes the main analysis, while the latter represents the exploratory analysis. The primary outcome (endpoint) of the study was progression-free survival (PFS) at the conclusion of the 4-year observation period, with disease control rate (DCR) at 1 year serving as secondary outcome (endpoint). The trial registration number for this study is NCT06629714.

### Participants

Eligible patients were screened and recruited from Oct 2020 to Apr 2024 at the Second Affiliated Hospital of Anhui Medical University. The inclusion criteria were as follows: (1) Meet the diagnostic criteria for esophageal, gastric or gastroesophageal junction (GEJ) cancer through clinical, pathological, and imaging examinations; (2) Karnofsky Performance Status (KPS) score should be at least 80, and the Eastern Cooperative Oncology Group Performance Status (ECOG PS) score should be no higher than 1; (3) Unresectable locally advanced or metastatic; (4) Systematic treatments naive (e. g., chemotherapy, anti-angiogenic drugs, targeted drugs, and immunotherapy); (5) Presence of at least one measurable lesion according to the Response Evaluation Criteria in Advanced Solid Tumors version 1.1 (RECIST v1.1); (6) Receiving PD-1/PD-L1 inhibitors monotherapy or combination with chemotherapy; (7) Informed and agreed to participate in the study; (8) Able to complete the questionnaire independently or with assistance from others if needed; (9) Legal age, 18 years or older.

Exclusion criteria were as follows: (1) Oncogene-driver positive (Example: Human Epidermal Growth Factor Receptor 2, HER-2); (2) Combined with other malignant tumors in the past 3 years; (3) Concurrent acute or chronic psychiatric disorders; (4) Current receiving anti-depressive or anti-anxiety therapy or other psychotropic drugs; (5) Previous treatment with other clinical drug trials; (6) Patients with symptomatic brain metastasis; (7) Severe intellectual disabilities or other communication difficulties that hindered normal interaction.

This study was conducted in accordance with the Declaration of Helsinki and was approved by the Ethics Committee of Anhui Medical University (83242393). Written informed consent was obtained from all participants prior to enrollment in the study.

### Treatment procedures

Treatment protocols using PD-1 and PD-L1 inhibitors, such as sintilimab, tislelizumab, camrelizumab, toripalimab, and pembrolizumab, were approved by China’s National Medical Products Administration for first-line therapy in advanced and inoperable GEC. The adjuvant chemotherapy regimen consisted of albumin-bound paclitaxel combined with platinum or fluorouracil combined with platinum. ICI were administered either as monotherapy or in combination with chemotherapy. Combination therapy was administered for durations of six to eight cycles, followed by maintenance treatment with ICI monotherapy. The maintenance therapy with ICIs continued for up to 2 years or until disease progression, death or unacceptable toxicity. Monotherapy with ICIs was administered for a duration of up to 2 years, or until disease progression, death, or unacceptable toxicity occurred.

### Data collection and evaluation

Baseline characteristics, including sociodemographic and radiographic information, were documented in an electronic case report form before immunotherapy initiation. We collected the sex information of participants determined by biological sex (female or male). Survival visits were performed every 3 months. PFS was defined as the duration between the date of initiation of ICIs and disease progression or death, whichever occurred first. The observation of PFS over a 4-year period, starting from the beginning and ending at the conclusion of the experiment, was the primary outcome of this study. DCR was defined as the proportion of patients achieving complete response (CR), partial response (PR), or stable disease (SD). The assessment of CR, PR, and SD was conducted in accordance with the RECIST v1.1 criteria. DCR of each patient who underwent ICIs therapy for a complete duration of 1 year was utilized as a secondary outcome measure.

### Evaluation of ED

ED commonly encompasses depression and anxiety symptoms^31,32^. Patient Health Questionnaire-9 (PHQ-9) and Generalized Anxiety Disorder 7-item Scale (GAD-7) were recommended in screening the depressive and anxiety symptoms in patients with cancer, respectively^32,33^.

PHQ-9 was the most widely used instrument for screening depression symptoms in primary health care^34^. Each of the 9 items was divided into four-point degrees of the scale (0 = not at all; 1 = some of the time; 2 = more than half the time; 3 = nearly every day) in the past two weeks. The total score ranged from 0 to 27. The PHQ-9 has been translated into Chinese and validated in clinical research, and the cutoff score of 5 demonstrated a favorable reliability and validity for screening depressive symptoms with high sensitivity and specificity in patients with cancer^35,36^.

GAD-7 is an effective tool for screening generalized anxiety disorder in clinical studies with good reliability and validity^37^. Each of the 7 items was rated on four-point degrees of the scale (0 = not at all; 1 = some of the time; 2 = more than half the time; 3 = nearly every day) in relation to the past two weeks. With a total score ranging from 0 to 21, a GAD-7 score ≥ 5 suggests the presence of anxiety symptoms^38^. GAD-7 was also translated into Chinese and had shown satisfactory validity and reliability for screening anxiety symptoms in patients with cancer^35,37^.

ED was determined by a sum of PHQ-9 and GAD-7 scores ≥10 according to validated thresholds in clinical practice^39,40^.

The score of ED was measured at baseline before initiation of ICI treatment. Patients responded to each item of the questionnaires and completed self-administered scales after a thorough explanation of the scale’s purpose.

### The collection and analysis of metabolic and inflammatory markers in peripheral blood

Metabolic and inflammatory markers were derived from the patients’ glucose, lipid profiles, and complete blood count conducted at the time of admission. All tests utilized peripheral venous blood samples, which were uniformly collected from patients starting at 8 am before the ICI treatment. The blood collection occurred after an overnight fast (with no food or water for 8 h), following a 15 min period of supine rest, and at least 12 h post abstinence from alcohol, coffee, and tobacco.

Metabolic markers, including blood glucose, triglycerides, total cholesterol, and albumin, alongside inflammatory cell counts (comprising absolute eosinophil count, eosinophil percentage, absolute neutrophil count (ANC), absolute lymphocyte count (ALC), absolute monocyte count (AMC), and absolute platelet count (APC)) were directly obtained from the examination. Certain inflammatory markers are calculated using the following formulas: NLR is the ratio of ANC to ALC; PLR is the ratio of APC to ALC; MLR is the ratio of AMC to ALC; SII is calculated as (ANC×APC)/ALC; PIV is equal to ANC×AMC×APC/ALC; PNI is equal to albumin level (g/L)  +  5 × ALC. We will employ absolute eosinophil count, eosinophil percentage, NLR, PLR, MLR, SII, PIV, and PNI as markers of systemic inflammation for our observations.

Metabolic and inflammatory markers were collected at two timepoints: prior to the baseline immunotherapy initiation (pre-treatment) and after two cycles of ICI treatment (post-treatment). Additionally, we assessed the relative changes in metabolic and inflammatory markers, specifically the ratio of these markers post-treatment to their levels pre-treatment (δ-treatment).

### Post hoc statistical power computation

Prior to study initiation, the absence of comparable studies precluded reliable sample size estimation through conventional power calculations. Conducting a pilot study was deemed impractical given the anticipated insufficient sample size (which would yield inadequate precision for power computation) combined with substantial resource constraints (including time and personnel). Consequently, we performed post hoc power analysis using PASS software (v11.0, NCSS). The multivariate Cox regression model for the primary endpoint demonstrated 91.7% statistical power, while the multivariate logistic regression analysis for secondary endpoints achieved 73.3% power. Detailed calculation process is provided in Fig S1-S2.

### Statistics and reproducibility

The characteristics of the ED group and the no ED group were delineated using categorical variables, presented as frequencies and percentages. Comparisons of baseline characteristics between groups were conducted using the *χ*2 test or Fisher’s exact test, as appropriate.

The association between ED and PFS was evaluated using Kaplan–Meier (K-M) survival curves and compared with the log-rank test. Univariate Cox proportional-hazards regression was used to estimate the association between ED and PFS. Multivariate Cox regression is employed to assess whether the association between ED and PFS remains significant after accounting for additional covariates, including all sociodemographic and clinical variables. To evaluate the association between ED and DCR, *χ*2 test was employed to compare the DCR between the two groups. Similarly, univariate logistic regression was used to estimate the association between ED and DCR. Multivariate logistic regression is employed to assess whether the association between ED and DCR remains significant after accounting for covariates. Sensitivity analyses assessed the associations between ED scores (sum of PHQ-9 and GAD-7), depression symptoms, anxiety symptoms, and individual depression and anxiety scores (PHQ-9 and GAD-7, respectively) with survival outcomes. Post hoc analysis examined the association between an alternative ED cutoff^40^ (defined as PHQ-9 ≥ 5 and/or GAD-7 ≥ 5) and survival outcomes.

The relationships between metabolic and inflammatory markers with primary and outcome constitute the exploratory analyses of this study. We established the receiver operating characteristic (ROC) curves with metabolic and inflammatory indicators as the test variables (X) and survival outcomes as the state variables (Y). Among them, since the survival outcome event at a specific time point cannot reflect the survival situation of the patient throughout the entire period, we defined that the PFS time of the patient being greater than or equal to the median PFS time of their ED group as a “favorable outcome”, and less than the median PFS time of their ED group as an “unfavorable outcome”. The favorable and unfavorable outcomes constituted the state variables of the ROC curves. The area under the curve (AUC) was calculated to evaluate the discriminatory power of the biomarkers. An AUC value with a 95% confidence interval (CI) excluding 0.5 was considered statistically significant. Biomarkers demonstrating statistical significance were screened out and included in subsequent analyses. The optimal cut-off values for these significant biomarkers were determined using the highest Youden’s index. Based on these cut-off values, patients were stratified into low-level and high-level groups for further survival analysis to assess clinical significance. K-M survival curves were plotted, and log-rank tests were conducted to compare survival differences between groups. Univariate and multivariate Cox proportional hazards regression models were employed to assess the associations between each biomarker and the primary outcome. While chi-square tests, univariate and multivariate logistic regression were employed to confirm and evaluate the associations between each biomarker and the secondary outcome.

Finally, the significant biomarkers identified in the aforementioned multivariate Cox regression analysis were respectively combined with ED to construct multivariate Cox regression models. These models were used to assess whether ED status and the levels of each biomarker independently affect the primary outcome after adjusting for other covariates. Similarly, the significant biomarkers identified in the aforementioned multivariate logistic regression analysis were respectively combined with ED to construct multivariate logistic regression models to assess whether ED and each biomarker independently affect the secondary outcome after adjusting for covariates. We also examined additive and multiplicative interactions between ED status and biomarker levels on survival events. The Mover method was employed to calculate the relative excess risk due to interaction (RERI), attributable proportion due to interaction (AP), and synergy index (SI) along with their 95% CI, given its superior performance in handling smaller sample sizes^41^. The results of Delta method were also reported as a sensitivity analysis. An additive interaction is indicated if the 95% CIs for RERI and AP do not include 0 and the 95% CI for SI does not include 1^42^. While multiplicative interaction was assessed using the interaction term in the regression model, with a 95% CI not including 1 indicating a significant multiplicative interaction.

**Fig. 1:**
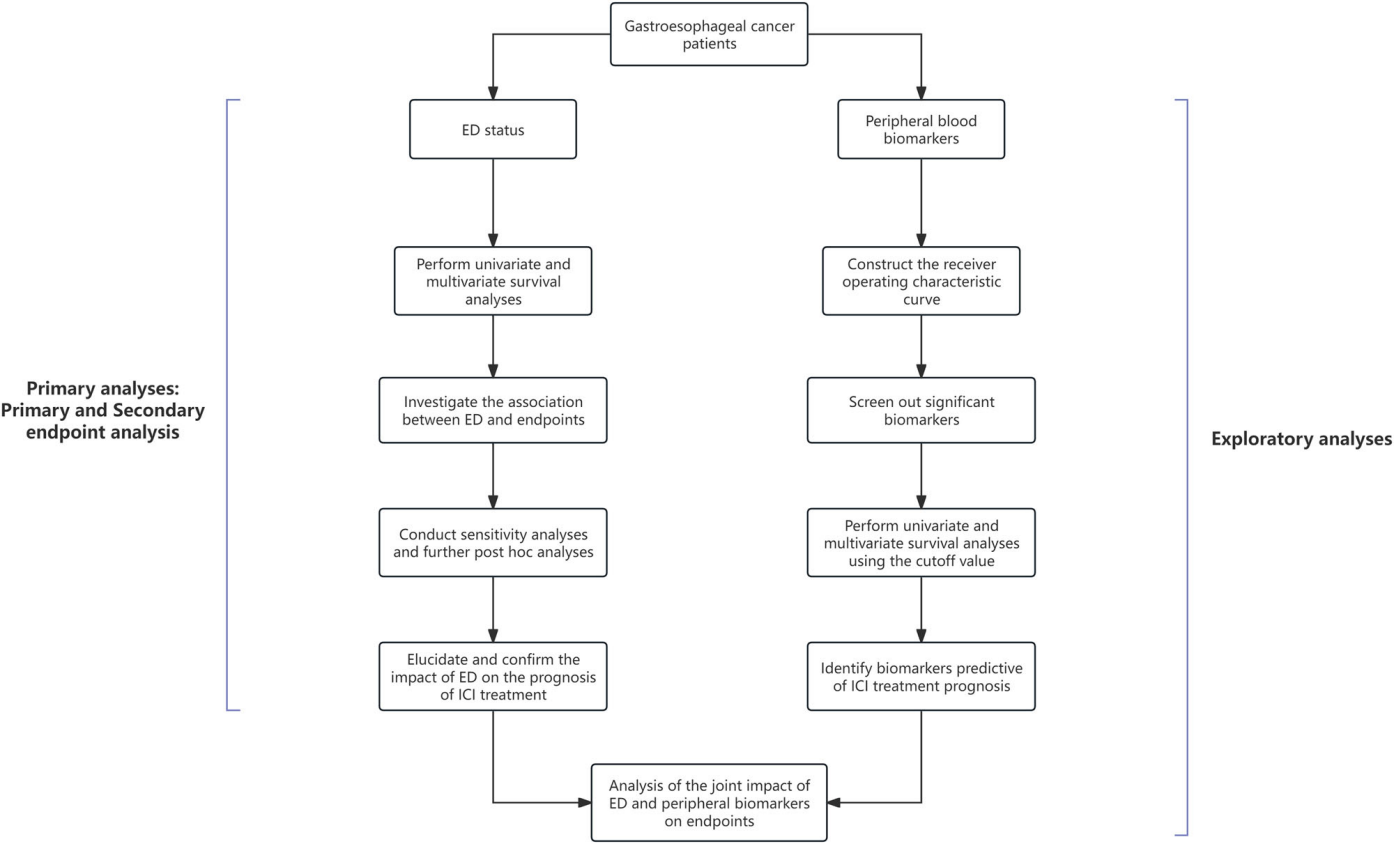
Methodological flowchart. The study comprised two main analytical approaches: a primary analysis and an exploratory analysis.

All statistical analyses were performed using SPSS 22.0 (IBM), R (v. 4.3.2), and Zstats 1.0 (www.zstats.net). The plots were generated using R (v. 4.3.2). In the univariate analysis, the research variables with *p* < 0.15 were included in the subsequent multivariate regression analysis model. In the multivariate regression model, *p* < 0.05 was considered statistically significant. Sociodemographic and clinical variables (Table A) were set as covariates in the multivariate analysis. Sensitivity analyses and post hoc analyses were using the same samples as those in the original test. All statistical tests were performed using two-sided significance testing. A comprehensive methodological flowchart is presented in Fig. 1.

## Results

### Population

**Fig. 2:**
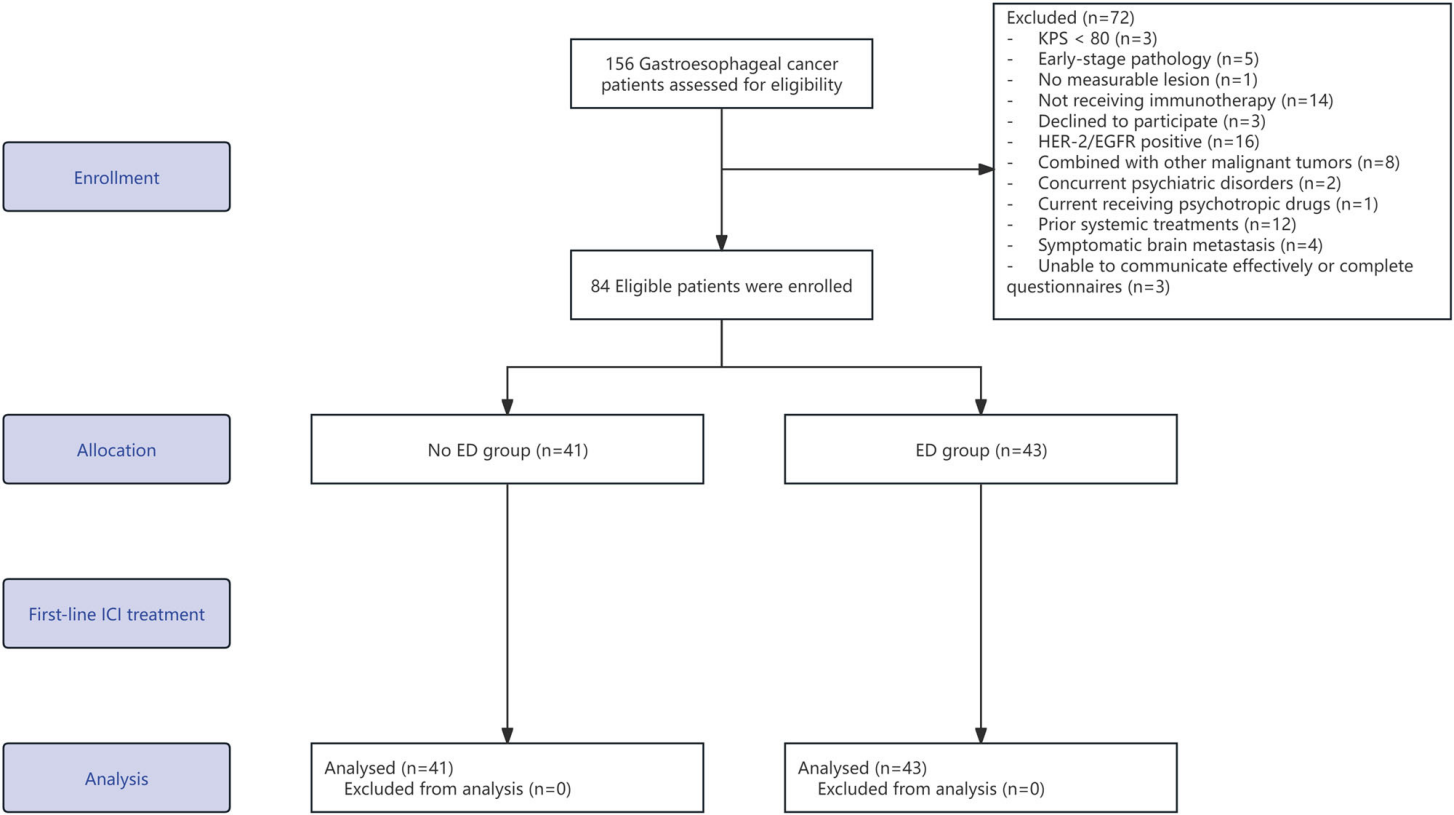
Flowchart diagram. A total of 156 patients with gastroesophageal cancer were assessed for eligibility. After applying the inclusion and exclusion criteria, 84 eligible patients were enrolled, allocated into two groups (No ED group, *n* = 41; ED group, *n* = 43), and subsequently entered a prospective observational follow-up study. All enrolled patients were included in the final analysis.

Between October 2020 and April 2024, 156 patients were screened, of whom 72 patients (46.2%) were excluded before enrollment; the main reasons for exclusion were Human Epidermal Growth Factor Receptor 2 (HER-2) or Epidermal Growth Factor Receptor (EGFR) positive, not receiving ICI treatment, and KPS score <80. A total of 84 patients with GEC were ultimately included for analysis in the study (Fig. 2).

We classified 84 patients into two groups according to their emotional status: ED group (n = 43) and no ED group (*n* = 41). Baseline sociodemographic and clinical characteristics of the participants were well balanced between the two groups (Table A). The study population predominantly comprised male patients (72.6%), individuals ≥65 years old (82.1%), and those who were unemployed or retired (73.8%), with a primary school education or below (63.1%), and married (91.7%). The primary post-admission caregivers were patients’ children (71.4%). A considerable proportion has a BMI below 24 (82.1%). The majority of patients were diagnosed with stage III and IV disease (84.5%), esophageal cancer (70.2%), and squamous cell carcinoma (71.4%); had an ECOG PS score of 0 (73.8%), a KPS score of ≥90 (73.8%), a CPS of ≥1 (71.4%) and a Visual Analogue Scale (VAS) score of 0 (61.9%); and received combination chemotherapy and immunotherapy (88.1%). Approximately 46.4% of the individuals were smokers or drinkers, while 50% had comorbidities of hypertension or diabetes. The mean time from pathological diagnosis to the ED assessment was 18.62 days (SD = 7.81), whereas the median time from the ED assessment to the initiation of immunotherapy was 3 days (IQR = 1.25−5.75). Furthermore, the specific treatment regimens for patients in both groups are detailed in Table S1, demonstrating a well-balanced distribution between the ED and no ED groups. Meanwhile, the tumor stages according to different primary tumor sites and the immunotherapy regimens across different pathological types are detailed in Table S2 and S3, respectively, with balanced distributions observed across all subgroups.

### Primary endpoint analysis: the association between ED and PFS

**Fig. 3:**
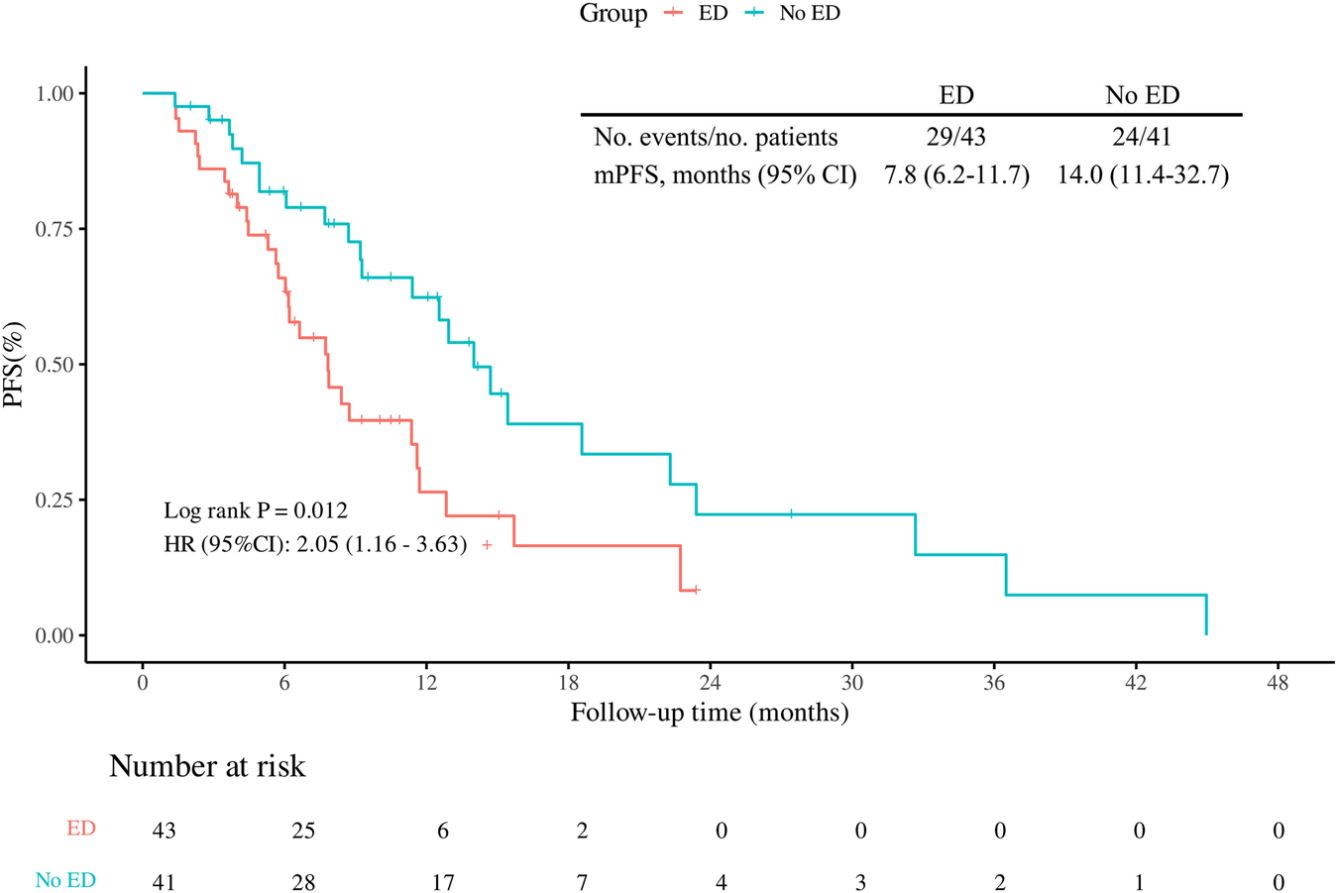
Kaplan–Meier curve of PFS by pre-treatment ED. No. number of, mPFS median PFS, CI confidence interval.

**Fig. 4:**
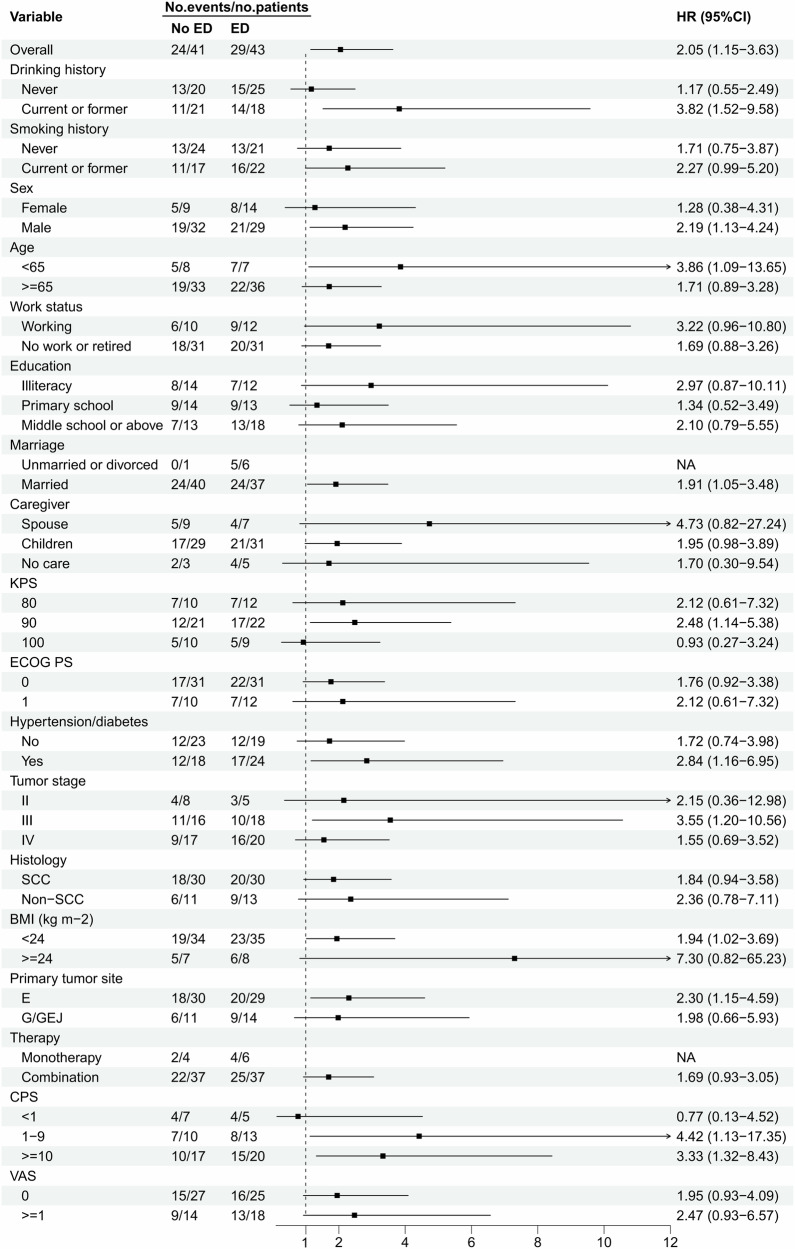
Subgroup analysis of PFS by pre-treatment ED. Hazard ratios and their corresponding 95% confidence intervals were derived from the Cox proportional hazards regression model, with all p-values computed using two-sided tests. Data are presented as the hazard ratios (the black square depicted in the figure) with error bars showing 95% confidence intervals (each thin line associated with every black square). No. number of, HR hazard ratio, CI confidence interval, Illiteracy: Possessing normal communication skills, but lacking the ability to comprehend written language; KPS Karnofsky performance status, ECOG PS Eastern Cooperative Oncology Group Performance Status, SCC Squamous cell carcinoma, Ade Adenocarcinoma, E Esophageal, G Gastric, GEJ Gastroesophageal junction, CPS Combined Positive Score, VAS Visual Analogue Scale. NA not applicable (insufficient sample size for reliable estimation).

With a data cutoff of August 31, 2024, the median follow-up time was 14.2 months (95% CI: 11.2–17.2), and a total of 53 PFS events were observed. The median PFS for the overall population was 11.4 months (95% CI: 8.2–14.6). The median PFS was 7.8 months (95% CI: 5.5–10.2) in the ED group and 14.0 months (95% CI: 11.1–16.9) in the no ED group. Log-rank testing revealed significantly shorter median PFS in the ED group compared to the no ED group (*χ²* = 6.26, *P* = 0.012). The survival curve generated using the K-M method is presented in Fig. 3. Univariate Cox regression analysis revealed that patients in the ED group exhibited a significantly higher risk of disease progression compared to those in the no ED group (HR = 2.05, 95% CI: 1.16–3.63; *P* = 0.014). The negative association between ED and PFS was consistent across various subgroups (Fig. 4), including the subgroups based on age ( < 65 years vs. ≥ 65 years), CPS ( ≥ 1 vs. <1), marriage (married versus unmarried or divorced), KPS (90 vs. 80 and 100), BMI ( < 24 vs. ≥ 24), male, alcohol consumers, stage III, esophageal cancer and among others. In the multivariate Cox regression analysis, ED (HR = 2.59, 95% CI: 1.35–4.97; *P* = 0.004) emerged as independent predictors for worse PFS. On the contrary, CPS ≥ 10 (HR = 0.40, 95% CI: 0.17–0.95; *P* = 0.037) was identified as a favorable predictor for PFS (Table B).

Sensitivity analysis examining the relationship between ED scores and PFS revealed significant associations. Univariate Cox regression analysis showed that the ED score was significantly associated with survival outcomes (HR = 1.04, 95% CI: 1.01–1.07; *P* = 0.008). After adjusting for covariates, the multivariate Cox regression analysis remained statistically significant (HR = 1.05, 95% CI: 1.02–1.08; *P* = 0.002). In addition, we analyzed the role of depression and anxiety symptoms alone in diminishing the efficacy of ICIs. The depression symptoms were associated with shorter median PFS (Univariate Cox regression: HR = 1.97, 95% CI: 1.12–3.45, *P* = 0.018; Multivariate Cox regression: HR = 2.82, 95% CI: 1.51–5.25, *P* = 0.001), as were the anxiety symptoms (Univariate: HR = 1.75, 95% CI: 1.01–3.05, *P* = 0.047; Multivariate: HR = 2.09, 95% CI: 1.13–3.88, *P* = 0.019). Similar results were observed for depression scores (Univariate: HR = 1.05, 95% CI: 1.01–1.09, *P* = 0.023; Multivariate: HR = 1.06, 95% CI: 1.02–1.11, *P* = 0.007) and anxiety scores (Univariate: HR = 1.08, 95% CI: 1.01–1.15, *P* = 0.018; Multivariate: HR = 1.11, 95% CI: 1.03–1.19, *P* = 0.005).

Post hoc analysis using alternative reported cutoff scores demonstrated that ED was associated with inferior PFS (Univariate: HR = 1.62, 95% CI: 0.89–2.97, *P* = 0.114; Multivariate: HR = 2.24, 95% CI: 1.14–4.38, *P* = 0.019). The results of the sensitivity analysis and post hoc analysis of the primary endpoint are presented and summarized in Table S4.

### Secondary endpoint analysis: the association between ED and DCR

One-year DCR was 53.6% (95% CI: 42.4–64.5) in the overall population. The DCR was 68.3% (95% CI: 51.9–81.9) in the no ED group and 39.5% (95% CI: 25.0–55.6) in the ED group, indicating that the ED group had a significantly lower DCR (*χ²* = 6.98, *P* = 0.008). These findings were confirmed by both univariate (OR = 3.29, 95% CI: 1.34–8.09; *P* = 0.009) and multivariate (OR = 3.21, 95% CI: 1.29–7.98; *P* = 0.012) logistic regression analyses (Table C). The main analysis results regarding the association between ED and both PFS and DCR are detailed in Table 1.

**Table 1:** Associations between ED and clinical efficacy of ICI (summary of main results)

Outcome	No ED (n = 41)	ED (n = 43)
Primary endpoint		
PFS		
No. events/no. patients at risk (%)	24/41 (58.5)	29/43 (67.4)
Median survival time, month (95% CI)	14.0 (11.4-32.7)	7.8 (6.2–11.7)
Univariate Cox regression—HR (95% CI)	reference	2.05 (1.15-3.63)
Multivariate Cox regression—HR (95% CI)	reference	2.59 (1.35-4.97)
Secondary endpoint		
DCR		
CR + PR + SD, percentage (95% CI)	68.3 (51.9–81.9)	39.5 (25.0–55.6)
Univariate logistic regression—OR (95% CI)	reference	3.29 (1.34–8.09)
Multivariate logistic regression—OR (95% CI)	reference	3.21 (1.29–7.98)

All statistical tests were two-sided. Cox proportional hazards regression was used for PFS analysis; logistic regression was used for DCR analysis.

*No.* number of, *CI* confidence interval, *HR* hazard ratio, *OR* odds ratio.

Similarly, we also conducted sensitivity analysis. Logistic regression revealed a significant negative association between the ED score and DCR (Univariate: OR = 1.08, 95% CI: 1.02–1.13, *P* = 0.005; Multivariate: OR = 1.14, 95% CI: 1.06–1.24, *P* = 0.001). In addition, depression symptoms (Univariate: OR = 4.19, 95% CI: 1.67–10.54, *P* = 0.002; Multivariate: OR = 4.36, 95% CI: 1.70–11.21, *P* = 0.002) and anxiety symptoms (Univariate: OR = 2.61, 95% CI: 1.08–6.30, *P* = 0.034; Multivariate: OR = 2.46, 95% CI: 1.01–6.03, *P* = 0.048) were associated with lower DCR. Similar results were observed for depression scores (Univariate: OR = 1.10, 95% CI: 1.02–1.19, *P* = 0.011; Multivariate: OR = 1.20, 95% CI: 1.06–1.36, *P* = 0.004) and anxiety scores (Univariate: OR = 1.17, 95% CI: 1.04–1.31, *P* = 0.008; Multivariate: OR = 1.31, 95% CI: 1.11–1.54, *P* = 0.002).

Post hoc logistic regression analysis also revealed that the alternative cutoff value of ED was significantly associated with a poorer DCR (Univariate: OR = 2.67, 95% CI: 1.03–6.89, *P* = 0.043; Multivariate: OR = 5.49, 95% CI: 1.27–23.66, *P* = 0.022). The results of the sensitivity analysis and post hoc analysis of the secondary endpoint are presented and summarized in Table S5.

### The associations between peripheral biomarkers and endpoints exploratory analyses

The ROC curves showed the predictive effect of each metabolic and inflammatory biomarker on survival (Fig. S3). The AUC and 95% CI analysis revealed that pre-treatment MLR (Pre.MLR), post-treatment NLR (Post.NLR), PLR (Post.PLR), MLR (Post.MLR), SII (Post.SII), PIV (Post.PIV), and δ-treatment eosinophil fraction (δ eosinophil%), NLR (δ NLR), PLR (δ PLR), MLR (δ MLR), SII (δ SII), and PIV (δ PIV) were significant biomarkers, as their 95% CI of AUC did not include 0.5. The optimal cut-off values, specificity, and sensitivity for these significant biomarkers are summarized in Table S6.

**Fig. 5:**
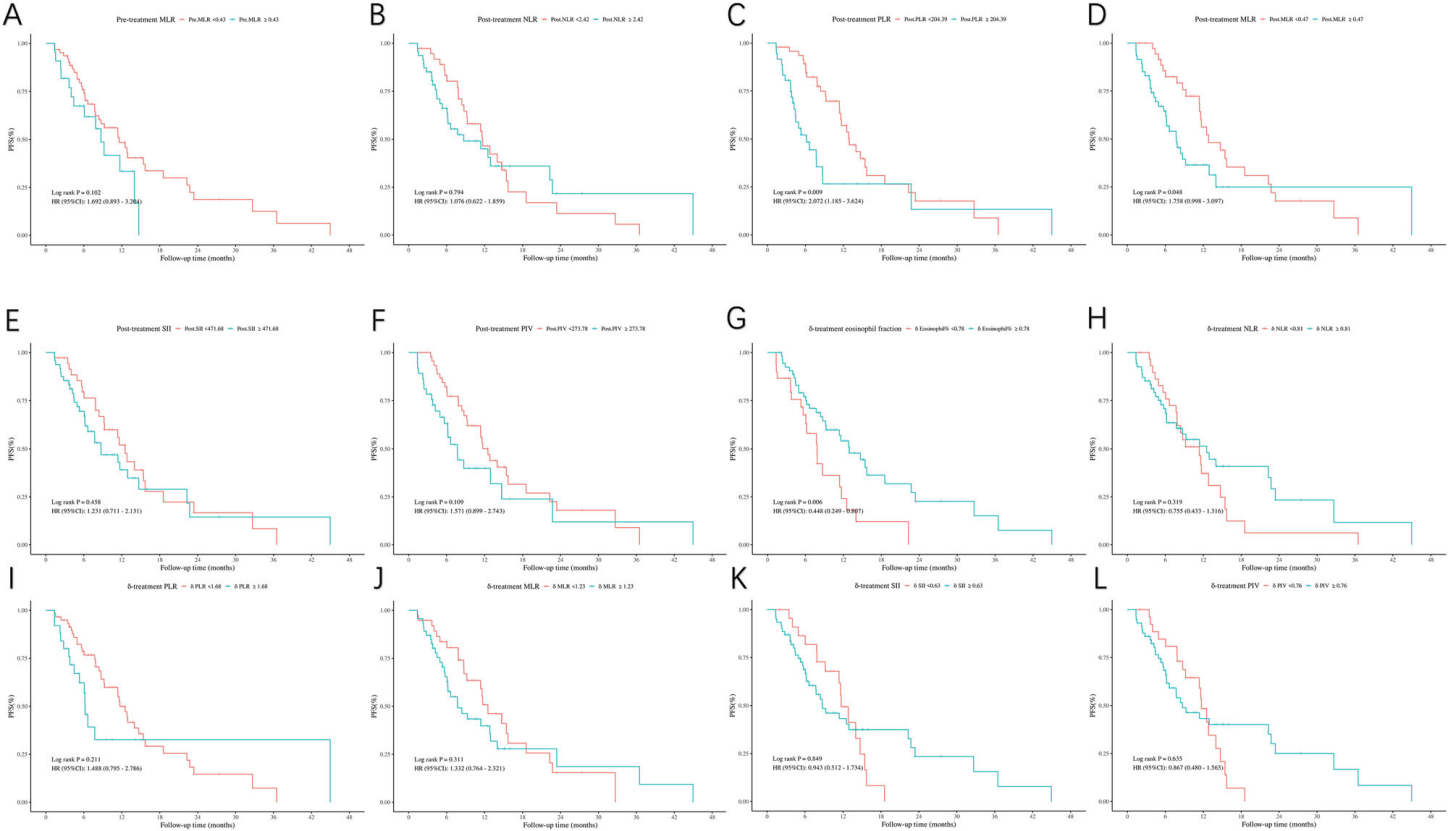
Kaplan–Meier curve of PFS by each significant biomarker. HR hazard ratio, CI confidence interval. **A** PFS by pre-treatment MLR ( < 0.43 versus ≥0.43), P-value from the log-rank test = 0.102, HR and 95% CI from univariate Cox regression = 1.692 (0.893–3.204); **B** PFS by post-treatment NLR ( < 2.42 versus ≥2.42), *P*-value from the log-rank test = 0.794, HR and 95% CI from univariate Cox regression = 1.076 (0.622–1.859); **C** PFS by post-treatment PLR ( < 204.39 versus ≥204.39), *P*-value from the log-rank test = 0.009, HR and 95% CI from univariate Cox regression = 2.072 (1.185–3.624); **D** PFS by post-treatment MLR ( < 0.47 versus ≥0.47), P-value from the log-rank test = 0.048, HR and 95% CI from univariate Cox regression = 1.758 (0.998–3.097); **E** PFS by post-treatment SII ( < 471.68 versus ≥471.68), *P*-value from the log-rank test = 0.458, HR and 95% CI from univariate Cox regression = 1.231 (0.711–2.131); **F** PFS by post-treatment PIV ( < 273.78 versus ≥273.78), P-value from the log-rank test = 0.109, HR and 95% CI from univariate Cox regression = 1.571 (0.899–2.743); **G** PFS by δ-treatment eosinophil fraction ( < 0.78 versus ≥0.78), *P*-value from the log-rank test = 0.006, HR and 95% CI from univariate Cox regression = 0.448 (0.249–0.807); **H** PFS by δ-treatment NLR ( < 0.81 versus ≥0.81), *P*-value from the log-rank test = 0.319, HR and 95% CI from univariate Cox regression = 0.755 (0.433–1.316); **I** PFS by δ-treatment PLR ( < 1.68 versus ≥1.68), P-value from the log-rank test = 0.211, HR and 95% CI from univariate Cox regression = 1.488 (0.795–2.786); **J** PFS by δ-treatment MLR ( < 1.23 versus ≥1.23), P-value from the log-rank test = 0.311, HR and 95% CI from univariate Cox regression = 1.332 (0.764–2.321); **K** PFS by δ-treatment SII ( < 0.63 versus ≥0.63), P-value from the log-rank test = 0.849, HR and 95% CI from univariate Cox regression = 0.943 (0.512–1.734); **L**. PFS by δ-treatment PIV ( < 0.76 versus ≥0.76), *P*-value from the log-rank test = 0.635, HR and 95% CI from univariate Cox regression = 0.867 (0.480–1.565).

Subsequently, the group survival analysis using the cutoff values of these significant biomarkers revealed that only the log-rank tests for pre-treatment MLR, post-treatment PLR, post-treatment MLR, and δ-treatment eosinophil fraction achieved statistical significance or approached significance (Fig. 5). Univariate and multivariate Cox regression analyses further substantiated these findings (Table D). All four biomarkers exhibited significant impacts on primary outcome.

To ensure the completeness of the analysis, we also examined the association between biomarkers and secondary outcome. The chi-square test demonstrated that the post-treatment PLR levels were significantly associated with DCR, whereas other biomarkers showed no significant correlation with DCR (Table S7). Univariate and multivariate logistic regression analyses further substantiated these findings (Table S8). The post-treatment PLR exhibited significant impacts on secondary outcome.

### The joint impact of ED and biomarkers on endpoints exploratory analyses

The multivariate Cox regression analysis revealed that (Table E-H), after adjusting for sociodemographic and clinical covariates, the four previously identified significant biomarkers could independently influence the primary survival outcome in conjunction with ED status. Specifically, patients with ED and elevated pre-treatment MLR, post-treatment PLR, and post-treatment MLR exhibited an increased risk of disease progression or death. Conversely, a higher δ-treatment eosinophil fraction was associated with a reduced risk of events. Interaction analyses revealed that ED and post-treatment PLR exhibited a significant multiplicative interaction effect on the primary survival outcome. Furthermore, while the Mover method identified a significant additive interaction, this finding was not confirmed by subsequent Delta method sensitivity analysis. In contrast, the other three biomarkers did not demonstrate either multiplicative or additive interaction effects with ED (Tables S9-12).

For the secondary outcome, the multivariate logistic regression analysis revealed that after adjusting for covariates, post-treatment PLR did not significantly influence the secondary survival outcome in conjunction with ED status (Table I). Elevated post-treatment PLR was associated with only a marginally significant increase in the risk of events (*P* = 0.061). Interaction analyses, both additive and multiplicative, between ED and post-treatment PLR indicated no significant interaction effects on the secondary survival outcome (Table S13).

## Discussion

This prospective cohort study demonstrates the dual impact of pretreatment ED and PIMs on ICI efficacy in treatment-naïve, advanced and inoperable GEC patients. Our findings demonstrate that baseline ED is associated with significantly shorter PFS and lower DCR. Furthermore, elevated pre-treatment and post-treatment MLR, post-treatment PLR, and reduced δ-treatment eosinophil fraction independently predict poorer immunotherapy outcomes. Importantly, our joint analysis revealed a significant multiplicative interaction between ED and post-treatment PLR, suggesting the combined psychological and inflammatory burden amplifies the risk of disease progression. These insights underscore the importance of integrating both psychosocial and biological markers into the management of advanced GEC and offer potential targets for enhancing therapeutic outcomes in this challenging patient population.

First, regarding the relationship between emotional factors and treatment outcomes, our findings demonstrate a robust relationship between baseline ED and adverse clinical outcomes, as reflected in reduced median PFS (7.8 months vs. 14.0 months) and DCR (39.5% vs. 68.3%) compared to no ED group. The ED group exhibited a 159% higher risk of disease progression compared to the no ED group, even after adjustment for potential confounders. Preclinical studies have demonstrated that stress factors, including emotional distress, can impair anti-tumor immune function by disrupting the homeostasis of the HPA axis, the sympathetic-adrenal-medullary (SAM) axis/SNS, and other neuroendocrine mechanisms. This disruption affects cellular signal transduction pathways and impairs the function of immune cells, potentially leading to reduced efficacy or treatment failure of ICIs. A study conducted by our team demonstrated that in chronic restraint stress-induced depressed mice with triple-negative breast cancer, plasma cortisol concentrations increased, CD8 + T cell numbers decreased, and the responses to PD-L1 inhibitor treatment were poorer, which were blocked by the cortisol-specific inhibitor metyrapone^43^. Another study conducted by other researchers demonstrated that standard temperature stress in mice enhanced β-adrenergic signaling, which reduced CD8 + T cell quantity and functionality while impairing PD-1 inhibitor efficacy, but these effects were reversed with the β-receptor blocker propranolol, though emotional symptoms were unexamined^44^. Our research from a clinical perspective demonstrates that ED significantly affects the efficacy and prognosis of GEC patients initially treated with ICI. This finding, supported by sensitivity analysis results, aligns with that of Zeng et al.^40^. Although their study focused on depressive or anxiety symptoms in non-small cell lung cancer (NSCLC) patients, whereas our study concentrated on depressive and anxiety symptoms in GEC patients. Notably, this is the first study to establish the significant impact of ED on the prognosis of GEC patients receiving ICI treatment.

In addition, dynamic changes in ED during cancer treatment may influence the outcomes of ICI therapy, although their prognostic value for survival appears less robust than that of baseline emotional status. For example, Zeng et al.^40^ reported that approximately 30% of patients with NSCLC experience shifts in emotional state during treatment. However, the ability of these longitudinal changes to predict treatment response and survival outcomes remains limited. Specifically, although patients whose distress improves tend to have better short-term prognosis compared to those with persistent distress, no significant difference in long-term survival is observed. Similarly, patients who develop new-onset distress do not exhibit significant differences in prognosis—particularly in the short term—compared to those who remain consistently free of distress. These findings suggest that baseline levels of ED may hold greater clinical relevance for survival prediction than intra-treatment emotional fluctuations. In this study, we did not systematically evaluate the temporal dynamics of emotional status during treatment, primarily because such changes may be confounded by tumor progression^45^, treatment-related adverse events^46^, and evolving systemic inflammation^26^. Our primary focus remains on assessing the impact of pre-treatment ED on ICI efficacy and patient outcomes. Nevertheless, future research should employ rigorous study designs, appropriate adjustment for covariates and potential confounders, and mechanistic investigations into emotional change to fully elucidate the role of ED and its trajectory in shaping treatment outcomes among cancer patients.

Beyond psychological factors, our analysis of peripheral inflammatory biomarkers revealed complementary prognostic insights. Our findings indicate that PIMs can predict the efficacy of immunotherapy in GEC patients. Specifically, a higher baseline MLR and elevated post-treatment PLR and MLR are associated with poorer PFS. Conversely, a greater increase in the eosinophil fraction after treatment compared to baseline is associated with improved PFS. The optimal cutoff values for these four PIMs were determined to be 0.43, 204.39, 0.47, and 0.78, respectively. These results are similar to those of a study by Huai et al.^47^ and two studies by Fu et al.^48,49^. However, the former study focused on the efficacy of neoadjuvant immunotherapy in patients with resectable NSCLC. In contrast, the latter two, despite involving patient populations similar to ours, were retrospective studies and only collected studies prior to the initiation of immunotherapy. Moreover, the PIMs assessed in these studies were not sufficiently diverse. For example, they did not account for changes in absolute eosinophil count and fraction, as well as the PNI. Our study prospectively explored whether these biomarkers before treatment, after two treatment courses (approximately 6 weeks), and their changes could predict the short-term efficacy (1 year) and long-term prognosis (4 years follow-up) of immunotherapy in advanced GEC patients. MLR and PLR may reflect an immunosuppressive tumor microenvironment characterized by lymphocyte-mediated immune impairment^50,51^. Conversely, eosinophils can enhance anti-tumor immune responses via cytotoxic effects and interactions with CD8 + T cells^52,53^, thereby exerting a protective role. These findings underscore the potential of PIMs to capture immunological changes associated with the efficacy of ICIs. Although the specific mechanisms remain unclear, these biomarkers provide clinicians with a non-invasive and readily accessible tool for evaluating patient responses to immunotherapy.

Our study revealed that post-treatment PLR uniquely demonstrated significant association with DCR. Specifically, a higher post-treatment PLR correlates with a lower DCR. This underscores the potential utility of continuous monitoring of PIMs to optimize treatment response prediction, particularly in later stages of ICI treatment. However, not all peripheral blood biomarkers exhibit significant correlations with DCR or PFS, such as metabolic indicators like albumin measured in this study. These metabolic biomarkers and metabolite-derived markers may be associated with overall survival in cancer patients, independent of specific treatments^29,54,55^. This highlights the heterogeneity in their prognostic value across different outcome measures and suggests the need for further investigation into their prognostic significance in specific therapeutic contexts.

Building upon these independent prognostic factors, analysis of ED and PIMs provided new insights into the complex interplay between emotional status and systemic inflammation in modulating immunotherapy outcomes. Initially, multivariate regression analysis revealed that ED and PIMs, particularly MLR, PLR, and eosinophil fraction, collectively influence the primary survival outcome. This finding further corroborates the results of previous regression analyses, indicating that ED and PIMs can independently affect ICI treatment efficacy while also coexisting to jointly impact treatment outcomes, especially long-term prognosis. In clinical practice, the patient’s emotional state and inflammation-related biomarkers should be carefully considered when evaluating immunotherapy efficacy or formulating treatment plans.

Further interaction analysis revealed a multiplicative interaction effect between ED and post-treatment PLR on the primary survival outcome. Additionally, the confidence intervals derived from the Mover method suggested an additive interaction, although this finding was not confirmed by sensitivity analysis using the Delta method. Collectively, these results indicate that the combined effect of ED and inflammation on survival outcomes exceeds their individual effects, suggesting a potential “synergistic” influence, particularly on long-term survival following ICI treatment. Studies have shown that ED (e.g., symptoms of depression and anxiety) can modulate systemic inflammation levels^27,56^, while elevated inflammation can exacerbate ED^57^, creating a self-reinforcing cycle that may synergistically impair immunotherapy efficacy beyond their isolated effects. This study is the first to identify this synergistic relationship. However, no significant interactions were observed between ED and other PIMs besides PLR, likely due to the limited sample size. Specifically, the Delta method, which typically requires larger sample sizes for statistical power^58^, failed to detect significant additive interactions between ED and PLR or other PIMs. Future studies with larger cohorts are necessary to further explore and fully elucidate the interplay between emotional status and inflammation in cancer prognosis.

These findings have important implications for clinical practice. The integration of psychological assessments and blood-based inflammatory biomarkers offers a dual-layered approach to predicting immunotherapy outcomes in GEC. Validated screening tools, including the PHQ-9 and GAD-7 can effectively identify patients at high risk for ED, enabling the implementation of targeted psychological and behavioral interventions before ICI initiation, such as effective and simple psychosocial therapies like the Managing Cancer and Living Meaningfully (CALM) intervention based on improving patients’ cognition and Behavioral Activation (BA) based on improving patients’ behavior^59,60^. Simultaneously, monitoring PIMs such as MLR and PLR at baseline and during early treatment can stratify patients based on predicted treatment efficacy. For those identified as high-risk (with PIMs at or above the validated cutoff), a comprehensive management strategy is recommended. This includes intensified monitoring of inflammatory markers, concomitant lifestyle interventions^61–63^ (e.g., smoking cessation, physical exercise, and an anti-inflammatory diet), and the judicious use of adjunctive anti-inflammatory medications^64^. Together, these strategies can serve as the foundation for personalized cancer care and treatment, where both physical and psychosocial factors are addressed.

This study provides the first comprehensive evaluation of associations between pretreatment ED, PIMs, and immunotherapy outcomes in advanced GEC. Its strengths include the robust methodological design, validated assessment tools, and prospective longitudinal data collection. However, there are several notable limitations. Firstly, the relatively small sample size of this study limited the statistical power for certain analyses, particularly subgroup analysis in survival analysis and interaction analysis in exploratory studies. Several subgroups in the survival analysis had insufficient sample sizes to generate reliable hazard ratio estimates or achieve statistical significance. Similarly, in the interaction analysis, the association between ED and certain PIMs did not reach statistical significance. Secondly, unmeasured confounding factors, such as TMB, which can influence the efficacy of ICI^65^, may have affected the observed associations. Moreover, the undetected dynamic trajectory of ED may potentially impact survival outcomes. Thirdly, the inclusion of multiple cancer types within GEC may limit the generalizability of the results to individual cancer types, although patients in this study received similar immunotherapies, and cancer type or histological subtype did not significantly impact survival in univariate and multivariate regression models. Finally, recruiting participants from a single center may restrict the generalizability of our findings to other geographic regions or healthcare settings. Future research should aim to validate these findings in larger-scale, more comprehensive, and multi-center cohorts and to dynamically explore interventions targeting ED and systemic inflammation as therapeutic adjuncts to improve ICI efficacy.

## Conclusions

This prospective cohort study demonstrated that pretreatment ED significantly impairs survival outcomes in patients treated with ICIs for advanced, treatment-naïve GEC, with ED patients showing substantially shorter PFS and lower DCR. The study identified key inflammatory biomarkers as significant predictors of immunotherapy response. Elevated pre-treatment MLR, post-treatment PLR, and MLR—whether independently or in combination with ED—were associated with poor outcomes. Conversely, increased eosinophil fraction dynamics were indicative of an improved response. Furthermore, a significant multiplicative interaction between ED and post-treatment PLR was observed, suggesting a synergistic effect on treatment outcomes.

These findings demonstrate the necessity of implementing a dual-monitoring approach in GEC patients receiving immunotherapy through systematic assessment of emotional well-being using validated screening tools and regular evaluation of peripheral inflammatory markers, particularly MLR and PLR. This strategy enables more precise risk stratification and treatment personalization, potentially improving immunotherapy outcomes. Furthermore, the identified interaction between psychological and inflammatory factors suggests that interventions targeting both pathways may synergistically augment the efficacy of ICI treatment in cancer patients.

## Supplementary information

Supplementary Information

Description of Additional Supplementary Files

Supplementary Data 1

Supplementary Data 2

## Data Availability

The source data underlying the figures are provided as Supplementary Data 2 alongside this manuscript. Specifically, the source data for Fig. 3 are available in “PFS by pretreatment ED”, for Fig. 4 in “PFS subgroup analysis” and for Fig. 5 in “PFS by each significant biomark”. Additional supporting data not included in Supplementary Data 2, as well as the complete datasets generated and/or analysed during this study, are not publicly available due to patient confidentiality requirements and institutional data protection policies. Deidentified individual patient-level clinical data are available under restricted access upon reasonable request. All requests for datasets should be directed to the corresponding author, CH (chd1975ay@126.com) and will be responded to within 2 weeks. Requests will be reviewed to determine whether the request is subject to any intellectual property or confidentiality obligations. Patient-related data requires the requesting researcher to sign a data access agreement, and data will be shared in aggregate form if there is no reasonable likelihood of participant reidentification.
