# Supplementary material for: Pretreatment emotional distress and peripheral biomarkers predict immune checkpoint inhibitor response in people with advanced inoperable gastroesophageal cancer
Source: Commun Med (Lond). 2026 Jan 26;6:154. doi: 10.1038/s43856-025-01358-9 (PMC13002868; doi:10.1038/s43856-025-01358-9)
Supplement: Supplementary file 1 — Supplementary Information [file 43856_2025_1358_MOESM1_ESM.pdf]

Table S1 Comparison of treatment regimens between the ED group and the No ED group

Characteristics	All (n=84)	No ED (n=41)	ED (n=43)	*P*
ICI treatment regimen, n (%)				0.869[a]
Sintilimab	50 (59.5)	25 (61.0)	25 (58.1)	
Tislelizumab	5 (6.0)	2 (4.9)	3 (7.0)	
Camrelizumab	14 (16.7)	8 (19.5)	6 (14.0)	
Toripalimab	11 (13.1)	5 (12.2)	6 (14.0)	
Pembrolizumab	4 (4.8)	1 (2.4)	3 (7.0)	
Chemotherapy regimen, n (%)				0.338
Nab-paclitaxel + Platinum	46 (62.2)	25 (67.6)	21 (56.8)	
Fluorouracil + Platinum	28 (37.8)	12 (32.4)	16 (43.2)	

Note: Chi-square tests were applied to all variables except those specifically marked, with all statistical p-values calculated using two-sided tests. [a] Calculated using Fisher's exact test.

Table S2 Comparison of tumor staging between esophageal cancer and gastric or gastroesophageal junction cancer

Characteristics	Total (n=84)	E[1] (n=59)	G[2]/GEJ[3] (n=25)	*P*
Tumor stage, n (%)				0.161
II	13 (15.5)	12 (20.3)	1 (4.0)	
III	34 (40.5)	22 (37.3)	12 (48.0)	
IV	37 (44.0)	25 (42.4)	12 (48.0)	

Note: Comparisons were performed using Chi-square test, with statistical p-values calculated using two-sided tests. [1] E: Esophageal; [2] G: Gastric; [3] GEJ: Gastroesophageal junction.

Table S3 Comparison of immunotherapy regimens between patients with squamous cell carcinoma and those with adenocarcinoma

Characteristics	Total (n=84)	Squamous (n=60)	Adenocarcinoma (n=24)	*P*
Combine chemotherapy or not				0.311[a]
No	10 (11.9)	9 (15.0)	1 (4.2)	
Yes	74 (88.1)	51 (85.0)	23 (95.8)	
ICI treatment regimen, n (%)				0.170[b]
Sintilimab	50 (59.5)	33 (55.0)	17 (70.8)	
Tislelizumab	5 (6.0)	3 (5.0)	2 (8.3)	
Camrelizumab	14 (16.7)	13 (21.7)	1 (4.2)	
Toripalimab	11 (13.1)	7 (11.7)	4 (16.7)	
Pembrolizumab	4 (4.8)	4 (6.7)	0 (0)	

Note: All statistical p-values calculated using two-sided tests. [a] Calculated using continuity-corrected Chi-square test; [b] Calculated using Fisher' s exact test.

Table S4 Summary of the sensitivity analysis and post hoc analysis investigating the association between ED and PFS

	Univariate Cox regression		Multivariate Cox regression[1]	
	P	HR (95% CI[2])	P	HR (95% CI)
ED scores	0.008	1.04 (1.01 ~ 1.07)	0.002**	1.05 (1.02 ~ 1.08)
Depression symptoms	0.018	1.97 (1.12 ~ 3.45)	0.001**	2.82 (1.51 ~ 5.25)
Anxiety symptoms	0.047	1.75 (1.01 ~ 3.05)	0.019*	2.09 (1.13 ~ 3.88)
Depression scores	0.023	1.05 (1.01 ~ 1.09)	0.007**	1.06 (1.02 ~ 1.11)
Anxiety scores	0.018	1.08 (1.01 ~ 1.15)	0.005**	1.11 (1.03 ~ 1.19)
Alternatively reported ED	0.114	1.62 (0.89 ~ 2.97)	0.019*	2.24 (1.14 ~ 4.38)

Note: Hazard ratios (HRs) and their corresponding 95% confidence intervals were derived from the Cox proportional hazards regression model, with all p-values computed using two-sided tests. [1] Adjusted for covariates; [2] CI, confidence interval; ED scores, the total score of PHQ-9 and GAD-7; Depression symptoms, the score of PHQ-9 ≥

5; Anxiety symptoms, the score of GAD-7 ≥ 5; Depression scores, the score of PHQ-9; Anxiety scores, the score of GAD-7; Alternatively reported ED, the score of PHQ-9 ≥ 5 and/or GAD-7 ≥ 5. * p<0.05; ** p<0.01.

Table S5 Summary of the sensitivity analysis and post hoc analysis investigating the association between ED and DCR

	Univariate logistic regression		Multivariate logistic regression[1]	
	P	OR (95% CI[2])	P	OR (95% CI)
ED scores	0.005	1.08 (1.02 ~ 1.13)	0.001**	1.14 (1.06 ~ 1.24)
Depression symptoms	0.002	4.19 (1.67 ~ 10.54)	0.002**	4.36 (1.70 ~ 11.21)
Anxiety symptoms	0.034	2.61 (1.08 ~ 6.30)	0.048*	2.46 (1.01 ~ 6.03)
Depression scores	0.011	1.10 (1.02 ~ 1.19)	0.004**	1.20 (1.06 ~ 1.36)
Anxiety scores	0.008	1.17 (1.04 ~ 1.31)	0.002**	1.31 (1.11 ~ 1.54)
Alternatively reported ED	0.043	2.67 (1.03 ~ 6.89)	0.022*	5.49 (1.27 ~ 23.66)

Note: Odds ratios (ORs) and their corresponding 95% confidence intervals were derived from the logistic regression model, with all p-values calculated using two-sided tests. [1] Adjusted for covariates; [2] CI, confidence interval; ED scores, the total score of PHQ-9 and GAD-7; Depression symptoms, the score of PHQ-9 ≥ 5; Anxiety symptoms, the score of GAD-7 ≥ 5; Depression scores, the score of PHQ-9; Anxiety scores, the score of GAD-7; Alternatively reported ED, the score of PHQ-9 ≥ 5 and/or GAD-7 ≥ 5. * p<0.05; ** p<0.01.

Table S6 The optimal cut-off values, specificity, and sensitivity of the significant biomarkers identified in ROC curves

**Significant biomarkers[a]**	**Best cut-off values**	**Specificity**	**Sensitivity**
Pre.MLR	0.43	0.89	0.33
Post.NLR	2.42	0.74	0.70
Post.PLR	204.39	0.85	0.56
Post.MLR	0.47	0.78	0.72
Post.SII	471.68	0.67	0.68
Post.PIV	273.78	0.81	0.56
δ Eosinophil%[b]	0.78	0.85	0.46
δ NLR	0.81	0.59	0.75
δ PLR	1.68	0.93	0.40
δ MLR	1.23	0.67	0.65
δ SII	0.63	0.56	0.82
δ PIV	0.76	0.56	0.77

Note: [a] The 95% confidence interval for the corresponding Area Under the Curve (AUC) does not encompass 0.5; [b]

δ-treatment eosinophil fraction. ROC, receiver operating characteristic; AUC, area under the curve. Statistical significance was determined by whether the 95% confidence interval for AUC excluded 0.5 (two-sided test).

Table S7 The chi-square test for the association between each significant biomarker identified in ROC curves and the secondary outcome

Biomarkers	CR+PR+SD, n (%)	PD, n (%)	Statistic	P
**Pre.MLR**			χ^2=0.79	0.374
Low level (<0.43)	35 (77.78)	27 (69.23)		
High level (≥0.43)	10 (22.22)	12 (30.77)		
**Post.NLR**			χ^2=0.01	0.937
Low level (<2.42)	20 (44.44)	17 (43.59)		
High level (≥2.42)	25 (55.56)	22 (56.41)		
**Post.PLR**			χ^2=5.46	0.019*
Low level (<204.39)	31 (68.89)	17 (43.59)		
High level (≥204.39)	14 (31.11)	22 (56.41)		
**Post.MLR**			χ^2=3.39	0.066
Low level (<0.47)	24 (53.33)	13 (33.33)		
High level (≥0.47)	21 (46.67)	26 (66.67)		
**Post.SII**			χ^2=0.57	0.449
Low level (<471.68)	21 (46.67)	15 (38.46)		
High level (≥471.68)	24 (53.33)	24 (61.54)		
**Post.PIV**			χ^2=0.64	0.422
Low level (<273.78)	27 (60.00)	20 (51.28)		
High level (≥273.78)	18 (40.00)	19 (48.72)		
**δ Eosinophil%**[1]			χ^2=1.97	0.161
Low level (<0.78)	13 (28.89)	17 (43.59)		
High level (≥0.78)	32 (71.11)	22 (56.41)		
**δ NLR**			χ^2=1.97	0.161
Low level (<0.81)	13 (28.89)	17 (43.59)		
High level (≥0.81)	32 (71.11)	22 (56.41)		
**δ PLR**			χ^2=1.31	0.252

Biomarkers	CR+PR+SD, n (%)	PD, n (%)	Statistic	P
Low level (<1.68)	34 (75.56)	25 (64.10)		
High level (≥1.68)	11 (24.44)	14 (35.90)		
**δ MLR**			χ^2=1.35	0.245
Low level (<1.23)	23 (51.11)	15 (38.46)		
High level (≥1.23)	22 (48.89)	24 (61.54)		
**δ SII**			χ^2=0.11	0.739
Low level (<0.63)	13 (28.89)	10 (25.64)		
High level (≥0.63)	32 (71.11)	29 (74.36)		
**δ PIV**			χ^2=0.06	0.802
Low level (<0.76)	15 (33.33)	12 (30.77)		
High level (≥0.76)	30 (66.67)	27 (69.23)		

Note: Comparisons were performed using Chi-square test, with statistical p-values calculated using two-sided tests.

[1] δ-treatment eosinophil fraction. *p<0.05.

Table S8 Univariate and multivariate logistic regression analyses of the association between each significant biomarker identified in ROC curves and the secondary outcome

Biomarkers	Univariate logistic regression		Multivariate logistic regression[a]	
	P	OR (95%CI[b])	P	OR (95%CI)
**Pre.MLR**				
Low level (<0.43)		1.00 (Reference)		
High level (≥0.43)	0.376	1.56 (0.59 ~ 4.14)		
**Post.NLR**				
Low level (<2.42)		1.00 (Reference)		
High level (≥2.42)	0.937	1.04 (0.44 ~ 2.46)		
**Post.PLR**				
Low level (<204.39)		1.00 (Reference)		1.00 (Reference)
High level (≥204.39)	0.021	2.87 (1.17 ~ 7.00)	0.030*	2.73 (1.10 ~ 6.74)
**Post.MLR**				
Low level (<0.47)		1.00 (Reference)		1.00 (Reference)
High level (≥0.47)	0.068	2.29 (0.94 ~ 5.55)	0.128	2.03 (0.82 ~ 5.06)
**Post.SII**				
Low level (<471.68)		1.00 (Reference)		
High level (≥471.68)	0.449	1.40 (0.59 ~ 3.35)		
**Post.PIV**				
Low level (<273.78)		1.00 (Reference)		
High level (≥273.78)	0.423	1.43 (0.60 ~ 3.39)		
**δ Eosinophil%[c]**				
Low level (<0.78)		1.00 (Reference)		
High level (≥0.78)	0.163	0.53 (0.21 ~ 1.30)		
**δ NLR**				
Low level (<0.81)		1.00 (Reference)		
High level (≥0.81)	0.163	0.53 (0.21 ~ 1.30)		
**δ PLR**				
Low level (<1.68)		1.00 (Reference)		
High level (≥1.68)	0.254	1.73 (0.67 ~ 4.45)		
**δ MLR**				
Low level (<1.23)		1.00 (Reference)		
High level (≥1.23)	0.247	1.67 (0.70 ~ 3.99)		
**δ SII**				
Low level (<0.63)		1.00 (Reference)		
High level (≥0.63)	0.739	1.18 (0.45 ~ 3.09)		
**δ PIV**				
Low level (<0.76)		1.00 (Reference)		
High level (≥0.76)	0.636	0.87 (0.48 ~ 1.57)		

Note: Odds ratios (ORs) and their corresponding 95% confidence intervals were derived from the logistic regression model, with all p-values calculated using two-sided tests. [a] Adjusted for sociodemographic and clinical covariates; [b] CI, confidence interval; [c] δ-treatment eosinophil fraction; *$p<0.05$.

Table S9 The multiplicative and additive interactions between ED and pre-treatment MLR on the primary survival outcome

	Mover method, OR (95% CI[1])	Delta method, OR (95% CI)
Multiplicative scale	1.45 (0.34 ~ 6.25)	1.45 (0.34 ~ 6.25)
RERI[2]	3.54 (-1.90 ~ 15.48)	3.54 (-2.72 ~ 9.80)
AP[3]	0.50 (-0.49 ~ 0.78)	0.50 (0.00 ~ 1.01)
SI[4]	2.43 (0.64 ~ 9.27)	2.43 (0.64 ~ 9.27)

Note: The Mover method and the Delta method were employed for primary and sensitivity analyses of interaction effects, respectively. [1] CI, confidence interval; [2] RERI, relative excess risk due to interaction; [3] AP, attributable proportion due to interaction; [4] SI, synergy index.

Table S10 The multiplicative and additive interactions between ED and post-treatment PLR on the primary survival outcome

	Mover method, OR (95% CI[1])	Delta method, OR (95% CI)
Multiplicative scale	4.25 (1.03 ~ 17.57)	4.25 (1.03 ~ 17.57)
RERI[2]	7.04 (1.93 ~ 19.99)	7.04 (-0.52 ~ 14.60)
AP[3]	0.79 (0.29 ~ 0.93)	0.79 (0.55 ~ 1.03)
SI[4]	8.67 (1.01 ~ 74.62)	8.67 (1.01 ~ 74.62)

Note: The Mover method and the Delta method were employed for primary and sensitivity analyses of interaction effects, respectively. [1] CI, confidence interval; [2] RERI, relative excess risk due to interaction; [3] AP, attributable proportion due to interaction; [4] SI, synergy index.

Table S11 The multiplicative and additive interactions between ED and post-treatment MLR on the primary survival outcome

	Mover method, OR (95% CI[1])	Delta method, OR (95% CI)
Multiplicative scale	1.92 (0.53 ~ 6.90)	1.92 (0.53 ~ 6.90)
RERI[2]	4.55 (0.16 ~ 15.68)	4.55 (-1.27 ~ 10.37)
AP[3]	0.61 (-0.02 ~ 0.84)	0.61 (0.25 ~ 0.96)
SI[4]	3.31 (0.91 ~ 12.03)	3.31 (0.91 ~ 12.03)

Note: The Mover method and the Delta method were employed for primary and sensitivity analyses of interaction effects, respectively. [1] CI, confidence interval; [2] RERI, relative excess risk due to interaction; [3] AP, attributable proportion due to interaction; [4] SI, synergy index.

Table S12 The multiplicative and additive interactions between ED and δ-treatment eosinophil fraction on the primary survival outcome

	Mover method, OR (95% CI[1])	Delta method, OR (95% CI)
Multiplicative scale	1.12 (0.30 ~ 4.26)	1.12 (0.30 ~ 4.26)
RERI[2]	-0.93 (-5.58 ~ 0.54)	-0.93 (-3.33 ~ 1.48)
AP[3]	-0.84 (-4.11 ~ 0.74)	-0.84 (-2.87 ~ 1.19)
SI[4]	0.10 (0.00 ~ 292.27)	0.10 (0.00 ~ 292.27)

Note: The Mover method and the Delta method were employed for primary and sensitivity analyses of interaction effects, respectively. [1] CI, confidence interval; [2] RERI, relative excess risk due to interaction; [3] AP, attributable proportion due to interaction; [4] SI, synergy index.

Table S13 The multiplicative and additive interactions between ED and post-treatment PLR on the secondary survival outcome

	Mover method, OR (95% CI[1])	Delta method, OR (95% CI)
Multiplicative scale	0.66 (0.10 ~ 4.54)	0.66 (0.10 ~ 4.54)
RERI[2]	1.54 (-12.30 ~ 18.29)	1.54 (-6.46 ~ 9.55)
AP[3]	0.22 (-1.98 ~ 0.70)	0.22 (-0.76 ~ 1.19)
SI[4]	1.34 (0.30 ~ 5.92)	1.34 (0.30 ~ 5.92)

Note: The Mover method and the Delta method were employed for primary and sensitivity analyses of interaction effects, respectively. [1] CI, confidence interval; [2] RERI, relative excess risk due to interaction; [3] AP, attributable proportion due to interaction; [4] SI, synergy index.

Cox Regression Power Analysis

Numeric Results

Power	Sample Size (N)	Reg. Coef. (B)	S.D. of X1 (SD)	Event Rate (P)	R-Squared X1 vs Other X's (R2)	Two-Sided Alpha	Beta
0.91734	84	0.9520	0.5000	0.6310	0.0670	0.05000	0.08266

References

Hsieh, F.Y. and Lavori, P.W. 2000. 'Sample-Size Calculations for the Cox Proportional Hazards Regression Model with Nonbinary Covariates', Controlled Clinical Trials, Volume 21, pages 552-560.
Schoenfeld, David A. 1983. 'Sample-Size Formula for the Proportional-Hazards Regression Model', Biometrics, Volume 39, pages 499-503.

Report Definitions

Power is the probability of rejecting a false null hypothesis. It should be close to one.
N is the size of the sample drawn from the population.
B is the size of the regression coefficent to be detected
SD is the standard deviation of X1.
P is the event rate.
R2 is the R-squared achieved when X1 is regressed on the other covariates.
Alpha is the probability of rejecting a true null hypothesis.
Beta is the probability of accepting a false null hypothesis.

Summary Statements

A Cox regression of the log hazard ratio on a covariate with a standard deviation of 0.5000 based on a sample of 84 observations achieves 92% power at a 0.05000 significance level to detect a regression coefficient equal to 0.9520. The sample size was adjusted since a multiple regression of the variable of interest on the other covariates in the Cox regression is expected to have an R-Squared of 0.0670. The sample size was adjusted for an anticipated event rate of 0.6310.

Fig. S1. The calculation of the statistical power for the multivariate Cox regression model of the primary endpoint

Note: X1 represents the variable of interest, which in this context is ED. Other X refers to the additional covariates included in the multivariate Cox regression model.

Logistic Regression Power Analysis

Numeric Results

Power	N	Pcnt N X=1	P0	P1	Odds Ratio	R Squared	Alpha	Beta
0.73268	84	51.200	0.317	0.598	3.210	0.015	0.05000	0.26732

References

Hsieh, F.Y., Block, D.A., and Larsen, M.D. 1998. 'A Simple Method of Sample Size Calculation for Linear and Logistic Regression', Statistics in Medicine, Volume 17, pages 1623-1634.

Report Definitions

Power is the probability of rejecting a false null hypothesis. It should be close to one.
N is the size of the sample drawn from the population.
P0 is the response probability at the mean of X.
P1 is the response probability when X is increased to one standard deviation above the mean.
Odds Ratio is the odds ratio when P1 is on top. That is, it is [P1/(1-P1)]/[P0/(1-P0)].
R-Squared is the R2 achieved when X is regressed on the other independent variables in the regression.
Alpha is the probability of rejecting a true null hypothesis.
Beta is the probability of accepting a false null hypothesis.

Summary Statements

A logistic regression of a binary response variable (Y) on a binary independent variable (X) with a sample size of 84 observations (of which 49% are in the group X=0 and 51% are in the group X=1) achieves 73% power at a 0.05000 significance level to detect a change in Prob(Y=1) from the baseline value of 0.317 to 0.598. This change corresponds to an odds ratio of 3.210. An adjustment was made since a multiple regression of the independent variable of interest on the other independent variables in the logistic regression obtained an R-Squared of 0.015.

Fig. S2. The calculation of the statistical power for the multivariate logistic regression model of the secondary endpoint

Note: X1 represents the variable of interest, which in this context is ED. Other X refers to the additional covariates included in the multivariate logistic regression model.

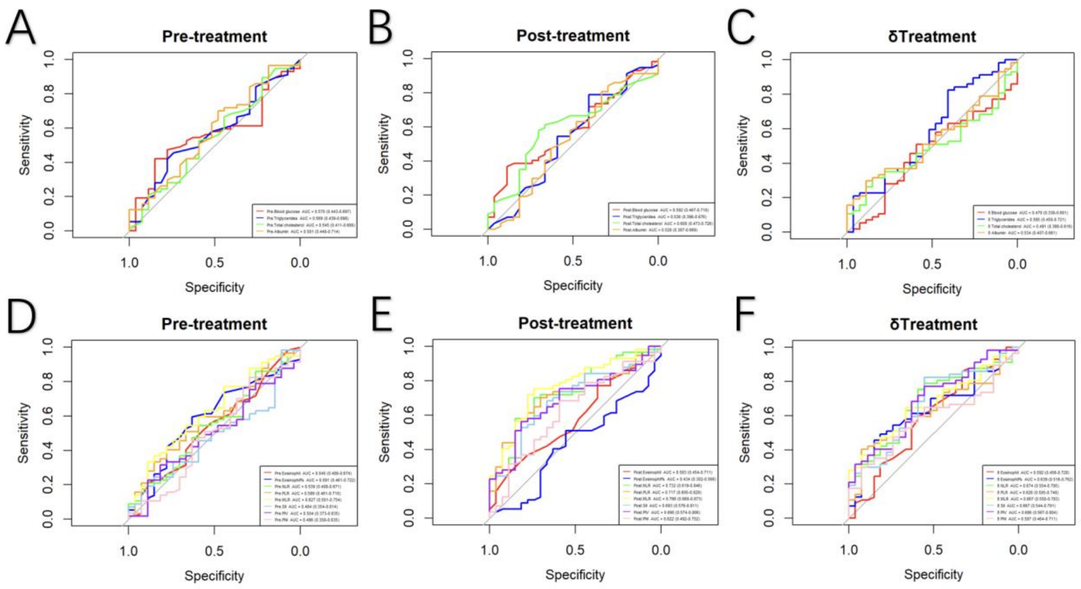

Fig. S3. The predictive effect of metabolic and inflammatory biomarkers on survival

Note: Figures A-C represent metabolic biomarkers, while figures D-F are inflammatory biomarkers; The legend presents the area under the ROC curve (AUC) and its 95% confidence interval for each biomarker
