# Supplementary material for: Pretreatment emotional distress and peripheral biomarkers predict immune checkpoint inhibitor response in people with advanced inoperable gastroesophageal cancer
Source: Commun Med (Lond). 2026 Jan 26;6:154. doi: 10.1038/s43856-025-01358-9 (PMC13002868; doi:10.1038/s43856-025-01358-9)
Supplement: Supplementary file 2 — Description of Additional Supplementary Files [file 43856_2025_1358_MOESM2_ESM.pdf]

Description of Additional Supplementary Files

Supplementary Data 1: Table A-I (comprising a total of 9 tables)

Supplementary Data 2: The source data for Fig. 3 are available in "PFS by pretreatment ED", for Fig. 4 in "PFS subgroup analysis", and for Fig. 5 in "PFS by each significant biomarker".
